# Fixing carbon credits requires a new financing model

**DOI:** 10.1093/pnasnexus/pgag117

**Published:** 2026-05-12

**Authors:** Benedict S Probst, Florian Egli

**Affiliations:** Net Zero Lab, Max Planck Institute for Innovation and Competition, 80333 Munich, Germany; Group for Sustainability and Technology, ETH Zurich, 8006 Zurich, Switzerland; Centre for Energy, Environment and Natural Resource Governance, Department of Land Economy, University of Cambridge, Cambridge CB2 1RX, United Kingdom; School of Social Sciences and Technology, Technical University of Munich, 80333 Munich, Germany; School of Management, Technical University of Munich, 80333 Munich, Germany

## Abstract

Carbon-crediting mechanisms could play a critical role in achieving net zero, yet growing evidence shows that many offset projects lack environmental integrity. Achieving geological net zero requires balancing residual fossil fuel–based emissions with permanent carbon dioxide removal (CDR), making the scale-up of CDR essential. However, current discussions on improving carbon-crediting mechanisms have focused too narrowly on implementation challenges, such as refining standards or monitoring systems. We argue that scaling permanent carbon removal requires a new financing model to address market barriers. This financing model must reduce price volatility and raise credit prices to attract investment. We propose a tiered auction framework to build and scale markets for novel CDR technologies by (i) setting a permanent removal target, (ii) ensuring minimum quality standards, and (iii) running reverse auctions combined with first-of-a-kind finance.

## Introduction

Carbon pricing is a central approach to mitigating climate change (1). Carbon-crediting mechanisms, a form of carbon pricing, allow project developers to earn carbon credits through voluntary and third party–certified mitigation projects such as forest protection or renewable energy (RE) projects (2). Private and public entities purchase these credits to underpin their emission reduction and net-zero strategies. Depending on the buyer, these carbon-crediting mechanisms have operated at different levels, ranging from international organizations, such as the Clean Development Mechanism (CDM), to nongovernmental entities like Verra. The CDM was used to meet Kyoto Protocol targets, whereas Verra-certified credits are used by governments, firms, nongovernmental organizations or individuals to meet voluntary emission reduction goals via offsetting (2).

Carbon credits from these mechanisms serve two main functions: first, to support compliance obligations or national mitigation targets, and second, to enable voluntary offsetting by firms and individuals. While both share similar infrastructure, they differ in governance and credibility requirements. Our analysis focuses on carbon credits that contribute to firms’ compliance obligations or national net-zero goals, for which robust financing mechanisms and regulatory oversight will be essential.

Despite their prevalence, carbon-crediting mechanisms have systematically failed to deliver the promised emission reductions. A recent systematic assessment documented that at least 84% of carbon credits across six central project types did not constitute real emissions reductions (2). Even carbon-crediting projects that achieve the claimed emissions reductions are often subject to large reversal risks. For instance, a review of forestry projects indicated that they systematically underestimate reversal risks (3). Most carbon credits today stem from temporary emission avoidance or emission reduction activities, such as forestry or improved cookstoves (4–6). Yet, natural climate solutions can play a meaningful role in future high-integrity carbon markets if market design is improved and permanence is properly priced (7).

To counter flaws in carbon-crediting mechanisms, current reform initiatives have focused on incremental improvements to standards and monitoring systems (8, 9). While these reforms are important, they risk leaving unaddressed two important facets of carbon offsetting. First, existing credits largely stem from project types that only provide short-term reductions or removals (10). Fossil fuel–based emissions remain in the atmosphere for hundreds to thousands of years, requiring a true offset to guarantee the reduction or removal for the same amount of time (11, 12). In practice, this means returning the carbon to the geological carbon cycle through approaches such as direct air carbon capture and storage (DACCS). Second, these project types, which we henceforth refer to as permanent carbon removal, are fundamentally different from most current project types, as they require high up-front investments and face significant technical risks (13). These characteristics require tailored policy and financial support, as demonstrated by the extensive literature on previous technological change in the energy sector, such as renewable energy (14).

Our central contention is that incremental reforms will not suffice to bring permanent carbon removal to market. Only a fundamental revamping of the financing model to support carbon removal can bring about that change. Our framework is oriented toward a future world that is close to net zero, in which residual fossil emissions from hard-to-abate sectors must be balanced with permanent removals. Yet, testing and deploying these technologies must be ramped up from current low levels, as the expertise and infrastructure required for large-scale deployment cannot wait until net zero is within reach.

## Why carbon markets will not scale without financial innovation

Voluntary carbon-crediting mechanisms aim to drive investments into projects that mitigate climate change at the lowest cost. A market with a diverse ecosystem of project developers, standard bodies, verifiers, validators, financiers, and marketplaces has evolved to serve this goal (13).

Yet, carbon markets differ fundamentally from standard commodity markets, such as those for electricity or agricultural products, and this difference partly explains why voluntary carbon markets do not suffice to deliver credible emission reductions. Commodity markets efficiently allocate goods with private use value (e.g. electricity to power homes). At the same time, carbon credits often have no direct use value to the buyer unless there is a mandatory carbon price or reduction target.

In voluntary markets, carbon removal remains a public good, with three key implications. First, the “market” only exists because of regulation or voluntary commitments and ceases to exist without them. Second, the delivery of the “good” needs to be verified by a third party because both buyer and seller have an incentive to overstate delivery. This requires sophisticated verification and validation systems. While commodities typically have similar and measurable quality characteristics, carbon credit projects, in contrast, seemingly yield the same product (e.g. 1 ton of mitigated carbon) but differ fundamentally in terms of quality, such as the permanence of carbon mitigation and potential side effects of project activities (e.g. social and biodiversity benefits or harms) (13). Third, permanence must be guaranteed for hundreds to thousands of years, far beyond the relevant time horizon for private companies, which necessitates new forms of institutional oversight (12). These characteristics make carbon markets fundamentally different from classical commodity markets, requiring a regulatory and financial architecture tailored to those product characteristics.

In a system that requires sophisticated institutions to generate, measure, deliver, and verify the permanence of products with vastly differing characteristics, the drive of markets to find the least-cost mitigation options favors unrealistically optimistic projects that often generate low-quality credits (e.g. avoided deforestation) (2). Such a market, searching for the least cost of only seemingly homogeneous products with varying quality characteristics, is inefficient and risks jeopardizing the credibility of carbon credits altogether.

**Figure 1:**
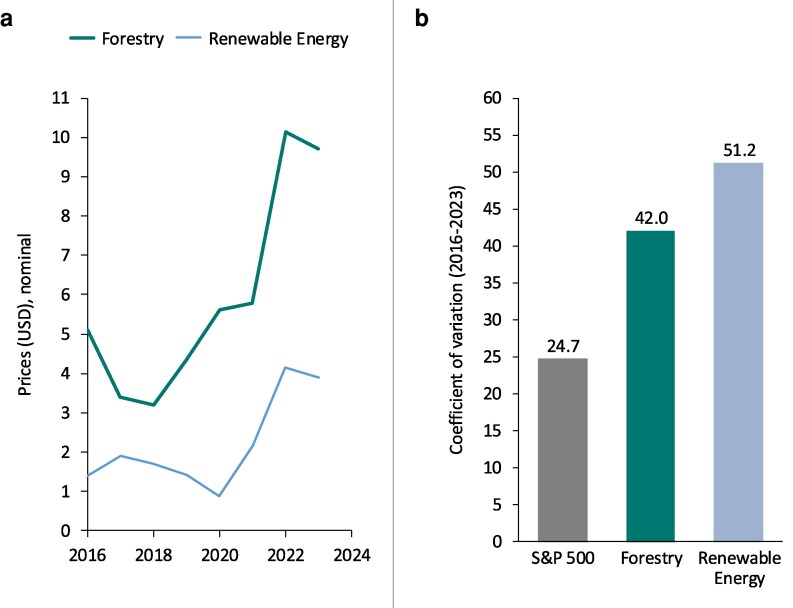
Price and volatility of carbon credits compared with standard financial assets (2016–2023). a) Average yearly carbon credit prices based on data from Ecosystem Marketplace (15) and b) volatility (calculated as the coefficient of variation from 2016 to 2023). Price volatility in the voluntary carbon market exceeded the S&P 500 over the observed period for forestry and renewable energy credits. Stocks are considered a relatively volatile asset class.

The current financing model relies on three streams of revenue. First, projects sell carbon credits into a voluntary market with low prices per ton and high volatility (see Fig. 1a and b). Second, projects that produce a good or a service beyond the sequestration of carbon sell into a market (e.g. electricity from wind energy projects). Third, many project developers receive subsidies and/or concessional finance to build and scale their business models.

**Figure 2:**
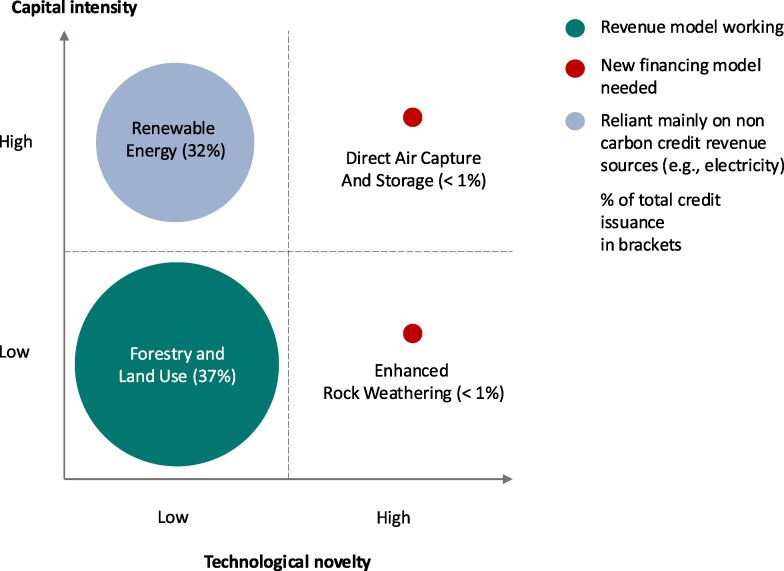
Carbon reduction and removal technologies. Technological novelty decreases as a dominant design emerges, technical challenges are solved, and a track record of manufacturing and deploying the technology in different settings is achieved. Our framework is oriented toward a future world that is close to net-zero, in which residual fossil emissions from hard-to-abate sectors must be balanced with permanent removals. Yet, testing and deploying these technologies must be ramped up from current low levels, as the expertise and infrastructure required for large-scale deployment cannot wait until net-zero is within reach. Capital intensity refers to the ratio of a technology's capital expenditure (CAPEX) to operating expenditure (OPEX). The current voluntary carbon market can be split into two categories: legacy and novel carbon removal. The legacy market primarily comprises credits from renewable energy, forestry, waste gas abatement, and cookstoves (2, 16). The novel carbon removal market—constituting <1% of the overall volume of issued credits—typically comes from less mature but potentially more permanent carbon removal methods (17). Typically, credits with low-technological novelty are traded in the legacy market, while methods with high technological novelty are traded in the carbon removal market. Smaller project categories were excluded, such as household and community projects (e.g. cookstoves) and chemical processes (e.g. SF6 destruction); therefore, the percentages do not add up to 100%. Based on cdr.fyi (17) (for DACCS and enhanced rock weathering) and Berkeley Carbon Trading Project Database (16) for forestry and land use, as well as renewable energy (American Carbon Registry, Climate Action Reserve, Gold Standard, and Verified Carbon Standard but excludes CDM and Joint Implementation due to differences in reporting.

To structure the discussion, we distinguish project types along capital intensity and technological novelty. Projects with higher capital intensity have comparatively higher upfront costs compared with operating costs, an example of which is renewable energy technologies (14). This capital structure makes them particularly sensitive to financing costs, whereas projects that accrue more operating costs are less sensitive to financing costs. Technological novelty reflects the level of risk related to technology performance, social acceptance, regulatory frameworks, and other contextual factors, which are uncertain if technologies have not been deployed for a long time and at scale. As such, technological novelty is associated with higher planning and implementation risk, translating into investment risk and higher financing costs. Based on these two categories, we propose four archetypical technology classes in Fig. 2, which also indicates their respective prevalence.

The most common carbon market project has low-capital intensity and low-technological novelty. Forestry and land use credits constitute 37% of voluntary carbon credits, mainly from projects seeking to avoid deforestation or improve forest management (16). A typical project in this category is avoiding deforestation, which has a low-capital intensity (e.g. limited up-front equipment required, most costs accrue during the project) and relies on proven technologies (e.g. monitoring forests and alternative livelihoods). Together with renewable energy credits, these make up for roughly 70% of the voluntary carbon market. This illustrates that the current market structure, favoring the cheapest credits, is unlikely to drive investments into novel technology projects. One of the reasons why more capital-intensive carbon dioxide removal (CDR) projects cannot proceed is that they are unable to access affordable financing for two key reasons.

First, exposure to revenue volatility makes financing, particularly debt financing, challenging, and, if possible at all, substantially increases the cost of capital, i.e. the financing cost. It is, therefore, unsurprising that studies indicate that revenues from carbon credits have seldom made renewable energy projects feasible (18, 19). Instead, the prevalence of renewable energy projects in carbon markets suggests that many of these benefit from a small revenue top-up via carbon credits but would have materialized without carbon credits too. Put differently, they rely on noncarbon credit revenues predominantly (Fig. 2).

Second, novel technologies are inherently riskier due to their lack of a performance track record (20). Investors want to see this increased level of risk matched with a higher return. Additionally, some novel technologies, such as DACCS, are around 100 times more expensive than current carbon credits (17). Hence, to support novel technologies, the cost delta needs to be compensated with higher levels of carbon credit prices, and the higher risk can only be addressed if the volatility of these credit prices decreases substantially compared with historical levels.

These patterns, including price volatility and technological risks, lead to market structures that systematically reward short-term, low-capital-intensity projects. This reinforces our central argument that scaling high-integrity, capital-intensive removal technologies requires a new financing model that stabilizes revenues and reduces financing costs. Early carbon removal startups, such as Climeworks or Running Tide, have relied on venture capital as equity risk capital to bridge the financing gap until credible offtake agreements or policy mechanisms can support bankable project finance. However, venture capital remains a limited and expensive source of finance that cannot sustain large-scale deployment. Similarly, demand for permanent carbon removal is extremely concentrated, as Microsoft alone accounted for >90% of durable removal purchases in 2025 (21). The financing and demand challenges faced by these firms illustrate structural constraints, namely the absence of stable, long-term demand and credible price signals.

## A new financing model

Despite market differences, evidence from other sectors, such as the development of renewable energy financing markets, can inform the choice of financing models for scaling novel and capital-intensive CDR technologies. Besides technical knowledge to develop and deploy first-of-a-kind projects, policies to reduce revenue risk were crucial for attracting capital, particularly lower-cost debt (14).

The most successful policies removed revenue risks entirely by guaranteeing a fixed offtake price for the entire projected lifetime of the projects. As RE technologies matured, supporting policies also became more elaborate. They moved to reverse auctions to elicit the market price by technology and remunerate at this level, and to contracts for difference to compensate only for the difference between the market price and a guaranteed level of revenue (14).

In this paper, we draw on the experience from renewable energy as a useful comparator to permanent carbon removal, not as a form of carbon credit. While the experience of renewable energy financing is instructive for policy design and sequencing, the analogy has limits. Renewable energy projects generate a tangible product (electricity) that can be sold in commercial markets, with policy instruments providing additional income. In contrast, CDR produces a public good and depends entirely on policy-created demand and verification. Financing models for CDR must therefore adapt these instruments to a market where revenue is generated solely from regulatory commitments, rather than from commodity sales. Despite these differences, renewable energy offers essential lessons for the design of CDR finance policies.

We therefore suggest similar policy actions and sequencing to scale permanent carbon removal. Given the state of the market, we recommend a tiered auction framework to build and scale markets for novel CDR technologies by (i) setting a permanent removal target, (ii) ensuring minimum quality standards, and (iii) running reverse auctions combined with first of a kind finance.

First, governments should have separate targets for gross emission reductions, temporary removals, and permanent removals (22). This differentiation ensures that it is clear how much of the net-zero goal is achieved by which sector and provides a credible scale-up path for permanent removals. The scale-up can inform the auctioned volumes that have to be procured by the governments or other actors over time. Second, governments should develop and enforce minimum standards, such as the Carbon Removal and Carbon Farming Framework in Europe. Third, the central element of our envisioned framework is a reverse auction mechanism that enables governments to procure CDR credits while allowing market forces to determine prices. Unlike fixed subsidies that risk overpaying, or market-based approaches that are often insufficient to drive funding to capital-intensive projects, our approach sits mid-way by allowing for price discovery while ensuring bankable revenue streams.

Inspired by renewable energy, governments can conduct regular CDR reverse auctions (e.g. semi-annual or yearly), as pioneered by Sweden and Denmark recently. The auction specifies a target volume to be procured by governments (e.g. X tonnes in 2027, rising to Y tonnes in 2035), and a ceiling price (differentiated by technology) that declines based on realized technology cost reductions (drawing on the innovation and learning curve literature). The latter is only necessary for technologies with few project developers to counter the risk of strategic bids in thin markets. Given that the demand from corporates is extremely narrow, in part due to its technical complexity and high cost (see above), we suggest that these auctions be open to corporates who wish to procure removals as well. Bids by project developers should be primarily evaluated on price, but may also include other co-benefits, such as job creation, technology transfer potential, or strategic independence.

The procured volume should have separate technology baskets to strike a balance between technology diversity and cost. The ceiling price for technologies with different durabilities could be set using an equivalence factor to value temporary storage relative to permanent storage (23). There is a trade-off in setting the number of auction baskets, as more baskets will lead to support for a more diverse set of removal technologies, including those that are currently too expensive to succeed in pooled auctions where a broad set of technologies can participate. However, too many baskets lead to thin markets where bidders have market power and can drive up costs, burdening public budgets. Hence, policymakers will need to choose carefully how technology-specific removal auctions will be. The contract duration should be longer for capital-intensive projects (e.g. DACCS, 15 years) and shorter for lower-capital-intensive technologies (e.g. biochar or enhanced rock weathering, 8–10 years). Payments should be structured along the verification milestones (e.g. quarterly).

In terms of cost allocation, we envisage three phases. In the first phase (e.g. 2025–2035), governments should bear the primary financial responsibility. The funding could either come directly from national budgets, from climate or innovation funds or from carbon tax revenues. The second stage (2035–2045), as technologies mature, responsibilities for financing should be increasingly shifted toward regulated emitters. This shift reduces pressure on fiscal budgets and signals the long-term commitment to scale beyond government support. The government's role would be to enact rules or quotas for removals for key emitters (similar to the government's own removal target) and to procure strategically important volumes in case of market shortfall. Third (2045+), in a more mature market phase, compliance with removal regulation should drive procurement, and the government's role becomes primarily regulatory (e.g. quality standards, registries, and storage oversight).

In addition, private-sector-led initiatives can complement government action and accelerate demand creation. Pooled private procurement initiatives, standardized long-term offtake contracts, and creditworthy intermediaries can help pool demand and enhance price discovery. Insurance and guarantee facilities can mitigate delivery and counterparty risks as private capital enters the market. Over time, corporate removal obligations and regulated demand quotas can sustain investment as direct public procurement phases out (see stages above).

Allocating risks appropriately ensures that taxpayers are not burdened unnecessarily while encouraging private investments. Technology and performance risk should be allocated to the project developers, as payout is tied to the successful removal. Put differently, if a project fails to deliver its envisaged reduction targets, the developer bears the financial responsibility. Yet, more systemic issues, such as fundamental safety issues for geological storage, could be covered by government insurance.

Yet many technologies, such as enhanced rock weathering, still face significant measurement uncertainties, and the underlying science will continue to evolve. It will therefore be impossible to set and enforce quality standards cleanly ex ante, and potential ex post adjustments threaten the credibility of the offtake agreement from the auction (24). Several potential remedies are conceivable. The proposed auction mechanism can draw on established systems, such as buffer pools, which designate a share of the issued credits to be set aside. The risk of adjustment could also be left to the project developers with certain limits, similar to the risk of technical nonperformance that renewable energy developers had to bear in early markets. Such an auction design could lead to the development of insurance-like instruments to pool the risk of overcrediting. Here too, we advocate for a staggered approach. In the early phase, governments should absorb most of the measurement risk, but as the science matures and uncertainty narrows, this risk can be gradually shifted to developers in successive auction rounds.

To complement the reverse auction mechanisms, governments can leverage an existing instrument at their disposal in many countries or regions. Public banks with deep engineering knowledge to develop and finance first of a kind (FOAK) projects should develop and finance early CDR projects, similar to what has been done for renewables (25). FOAK should exclusively focus on funding of CDR solutions with high capital intensity, such as direct air capture and storage (Fig. 2). A key hurdle for hard-tech climate startups, such as those developing CDR, is that financing is not available for large pilot and commercialization plants, i.e. FOAK. Public banks could establish dedicated funding lines and engineering teams for CDR FOAK, also in collaboration between banks. Experience with FOAK can help crowd in other financial actors, as evidenced in the renewables space. However, it will only work if there is a market for carbon removals (i.e. an offtake of the produced “product”). Therefore, FOAK financing need not be last in terms of policy action, but it will not function in isolation.

Finally, the essence of a carbon removal market is to store carbon dioxide, which will require payment without granting the right to use it, as markets commonly do. Scaling these markets will therefore remain challenging and require strong regulation, likely combined with sizeable subsidies. Whether such subsidies can be sustained politically over time is unclear, and sunset clauses for support schemes, such as the proposed auctions, can help. Such sunset clauses can be either strictly time-bound, with policy expiry dates aligned with the previously discussed time horizons. A smarter strategy may be to periodically evaluate the permanence and cost reductions of different removal technologies to phase out or adjust policy support in auction baskets (see above) with poor performance. Ensuring political acceptance is crucial because developing a carbon removal market with low- and high-volatile offtake prices will not succeed because of high financing costs. The choice will be to either commit substantial resources and policy attention to building a solid carbon removal market with private finance participation or to accept another voluntary carbon credit market plagued by scandal and poor delivery, undermining confidence in the very concept of carbon markets.

## Supplementary Material

pgag117_Supplementary_Data

## References

[1] World Bank . State and Trends of Carbon Pricing 2023. 2023. [accessed 2025 May 5]. https://openknowledge.worldbank.org/handle/10986/39796

[2] Probst B, et al 2024. Systematic assessment of the achieved emission reductions of carbon crediting projects. Nat Commun. 15:9562. 39543137 10.1038/s41467-024-53645-z PMC11564741

[3] Holm JA, Anderegg WRL, Bomfim B, So IS, Haya BK. Durability. In: Haya BK, editor. Quality assessment of REDD+ carbon credit projects. Berkeley Carbon Trading Project, 2023. p. 132–152.

[4] Gill-Wiehl A, Kammen DM, Haya BK. 2024. Pervasive over-crediting from cookstove offset methodologies. Nat Sustain. 7:191–202.

[5] West TAP, Börner J, Sills EO, Kontoleon A. 2020. Overstated carbon emission reductions from voluntary REDD+ projects in the Brazilian Amazon. Proc Natl Acad Sci U S A. 117:24188–24194. 32929021 10.1073/pnas.2004334117 PMC7533833

[6] West TA, et al 2023. Action needed to make carbon offsets from forest conservation work for climate change mitigation. Science. 877:873–877. 10.1126/science.ade3535 37616370

[7] Anderegg WRL, et al 2025. Towards more effective nature-based climate solutions in global forests. Nature. 643:1214–1222. 40739023 10.1038/s41586-025-09116-6

[8] ICVCM . *Core Carbon Principles*. ICVCM, 2024. [accessed 2025 Jun 7]. https://icvcm.org/the-core-carbon-principles/

[9] VCMI . Voluntary Carbon Markets Integrity Initiative. 2025. [accessed 2025 May 7]. https://vcmintegrity.org/

[10] Haya BK, et al *Voluntary registry offsets database v2025-04*. Berkeley Carbon Trading Project, University of California, Berkeley, 2025.

[11] Allen MR, et al 2025. Geological Net Zero and the need for disaggregated accounting for carbon sinks. Nature. 638:343–350. 39557072 10.1038/s41586-024-08326-8

[12] Brunner C, Hausfather Z, Knutti R. 2024. Durability of carbon dioxide removal is critical for Paris climate goals. Commun Earth Environ. 5:645.

[13] Smith S, et al The state of carbon dioxide removal. 2nd ed. University of Oxford, 2024.

[14] Polzin F, Egli F, Steffen B, Schmidt TS. 2019. How do policies mobilize private finance for renewable energy?—a systematic review with an investor perspective. Appl Energy. 236:1249–1268.

[15] Ecosystem Marketplace . *2024 State of the Voluntary Carbon Market (SOVCM)*. 2024. [accessed 2025 May 7]. https://www.ecosystemmarketplace.com/publications/2024-state-of-the-voluntary-carbon-markets-sovcm/

[16] Haya BK, Abayo A, Rong X, So IS, Elias M. *Voluntary registry offsets database v11*. Berkeley Carbon Trading Project, University of California, Berkeley, 2024.

[17] CDR.fyi. 2024. [accessed 2025 May 8]. https://www.cdr.fyi/

[18] Calel R, Colmer J, Dechezleprêtre A, Glachant M. 2025. Do carbon offsets offset carbon? Am Econ J Appl Econ. 17:1–40.

[19] Chan G . *Essays on energy technology innovation policy*. 2015. [accessed 2025 May 7]. https://dash.harvard.edu/entities/publication/73120379-2c57-6bd4-e053-0100007fdf3b

[20] Egli F . 2020. Renewable energy investment risk: an investigation of changes over time and the underlying drivers. Energy Policy. 140:111428.

[21] ClimeFi . CDR Market Review. 2025. [accessed 2025 March 7]. https://www.climefi.com/blog-posts/2025-cdr-market-review

[22] European Scientific Advisory Board on Climate Change . Scientific Advice for Amending the European Climate Law: Setting Climate Goals to Strengthen EU Strategic Priorities. 2025. [accessed 2026 Feb 7]. https://climate-advisory-board.europa.eu/reports-and-publications/scientific-advice-for-amending-the-european-climate-law-setting-climate-goals-to-strengthen-eu-strategic-priorities

[23] Groom B, Venmans F. 2023. The social value of offsets. Nature. 619:768–773. 37407820 10.1038/s41586-023-06153-x

[24] Cabiyo B, Field CB. 2025. Embracing imperfection: carbon offset markets must learn to mitigate the risk of overcrediting. PNAS Nexus. 4:1–4. 10.1093/pnasnexus/pgaf091 PMC12063487 40352644

[25] Geddes A, Schmidt TS, Steffen B. 2018. The multiple roles of state investment banks in low-carbon energy finance: an analysis of Australia, the UK and Germany. Energy Policy. 115:158–170.

